# Short-Term and Highly Intensive Early Intervention FIAS: Two-Year Outcome Results and Factors of Influence

**DOI:** 10.3389/fpsyt.2020.00687

**Published:** 2020-07-14

**Authors:** Evelyn Herbrecht, Olga Lazari, Marianne Notter, Esther Kievit, Klaus Schmeck, René Spiegel

**Affiliations:** Child and Adolescent Psychiatry, Psychiatric University Hospitals, University of Basel, Basel, Switzerland

**Keywords:** autism spectrum disorder, early intervention, FIAS, outcome, autism symptom severity

## Abstract

**Background:**

Comprehensive behaviorally or developmentally based early intervention programs have been shown to be effective in improving cognitive, social communicative, and adaptive skills of children with autism spectrum disorder (ASD). Besides the definition of relevant outcome predictors, the question of whether early intensive intervention positively changes core autism symptoms in children, as well as their long-term outcome, is an important issue for current research. The primary objective of the current study was to examine whether symptomatic and behavioral changes in children up to 4.5 years with ASD were sustained one and two years after an initial 18 days of intensive FIAS (Frühintervention bei autistischen Störungen) intervention.

**Methods:**

We analyzed the data of 32 young children with moderately severe to severe ASD who had been treated at the FIAS center between January 2011 and July 2017 and who had completed their 2-year follow-up in summer 2019.

**Results:**

ADOS total scores decreased significantly from baseline to the 1-year follow-up and from baseline to the 2-year follow-up (p < 0.01), with the most prominent change being from baseline to 2-year follow-up. The DD-C-GAS, a global scale used to assess four areas of everyday functioning, showed highly significant improvements on all subdomains. We found mostly significant correlations between results on both rating instruments at all time points, yet mostly no meaningful correlation between their changes over time. There was a close and statistically significant relationship between parents’ treatment adherence and ADOS scores, indicating that the better parents’ treatment adherence, the lower the children scored on the ADOS at 1- and 2-year follow-up. Overall, improvement on both scales was virtually independent of age and autism symptom severity at baseline, suggesting that older (>43 months) and more severely affected children (ADOS total score >20) may benefit from the FIAS intervention to the same extent as younger children do.

**Conclusions:**

The results of the study indicate that the FIAS approach of providing an initial highly intensive 18-day intervention period, followed by 2 years of less intensive follow-up care had an impact on the core autism symptoms as well as the adaptive functioning of children with ASD.

## Introduction

Since 2011, we have applied FIAS (Frühintervention bei autistischen Störungen), a highly intensive treatment method derived from the Mifne approach (1), to young children with moderately severe to severe autism spectrum disorder (ASD). FIAS is a play-based early intervention used with children aged up to 5 years. Its aim is to encourage children to get involved in social interactions by increasing their shared enjoyment and social motivation. A distinctive feature of FIAS is an initial 18-day period of very intensive treatment of the child, together with his/her core family, in a specialized unit, followed by continued, professionally supervised, play interaction between the parents and the child in the family home.

Evidence from early intensive intervention research points to the effectiveness of comprehensive, behaviorally or developmentally based intervention programs on cognitive, social communication, and language functioning as well as adaptive skills (2–4). Outcomes across different studies have often shown significant treatment effects, albeit with usually small effect sizes (5); moreover, outcomes have generally been found to vary across studies, reflecting the heterogeneity of patient profiles, specific treatment approaches, and the diversity of outcome measures used (6). Most early intervention studies published in recent years have found pre-treatment intellectual ability, as well as language and adaptive behavior abilities, to be the most reliable predictors of positive outcomes (7). Conversely, the severity of autism symptoms and age at the start of intervention have shown rather inconsistent results in terms of outcome predictability (8). However, there is some evidence that interventions targeted at improving social communication may be most effective at around the age of 3-1/2 years (3).

The duration of most early interventions in the studies that have been published varies from between 1 and 2 years of intensive intervention. However, there is insufficient evidence concerning the role of intervention duration (4). Fossum et al. (9) showed that, standard measures of pre-treatment cognitive and language abilities aside, positive affect and level of appropriate toy contact significantly influenced children’s expressive language outcome after one year of a community “Pivotal Response Treatment” program. In addition, less social avoidance and fewer repetitive vocalizations were among the larger benefits of the intervention. Variability in intervention outcomes has been repeatedly shown across different intervention approaches; and yet the sources of this variability are unclear, and whether and which developmental profiles benefit most from which kind of early intervention approach remain crucial questions (3, 9). In addition to identifying relevant outcome predictors, the question of whether early intensive intervention positively changes the long-term outcomes for children is another important issue in current research, as most studies have focused on outcomes immediately following the end of the intervention. Magiati et al. (4), who summarized a number of reviews and meta-analyses, concluded that children continue to make progress in terms of standard measures of cognitive and adaptive behavior after the end of an intensive intervention period. However, they also suggested that such gains often decrease during follow-up and argued that there is currently no evidence to suggest that early interventions reduce the need for special support with respect to long-term outcomes.

In a previous study (10) we reported positive short-term changes both in specific autism-related behaviors and in everyday functioning domains in 40 children after an 18-day period of FIAS early intervention. Changes in autism symptoms were captured by the Autism Behavior Coding System (ABCS), a video-based instrument to assess core autism symptoms during therapist-child interaction. We found the most relevant improvement in social cooperative behavior, expression of wishes, and eye contact. Additionally, the study reported on highly significant improvement of everyday functioning domains on the Developmental Disorders–Child–Global Assessment Scale (DD-C-GAS).

The present study set out to examine whether an early intensive FIAS intervention showed effects 1 and 2 years after the initial intervention period, by considering the following questions: (a) are the effects of FIAS interventions reflected in independently rated standard assessments of autism symptoms [(using the Autism Disorder Observation Scale or ADOS-2) (11); German version (12)] at 1 and 2 years after intervention? (b) is there a relationship between age and autism symptom severity at baseline and ADOS score changes after 1 and 2 years? (c) to what extent are the effects of FIAS interventions reflected in assessments made by clinicians using the DD-C-GAS? (d) what is the relationship between the ADOS and DD-C-GAS results? (e) is there a relationship between either the ADOS or DD-C-GAS results and parents’ treatment adherence during the 2 years of follow-up care?

## Materials and Methods

### The FIAS Approach

FIAS treatment consists of an initial 18-day intervention period with the core family at the FIAS center involving up to 6 h of play sessions per day with alternating therapists. Parents are intensively coached while playing with their child themselves and while observing their child’s behavior *via* direct and video observation of therapist–child interactions during play sessions. Sessions with the child are relation-oriented and include exploration, imitation, functional, and symbolic play as well as everyday activities as eating, clothing, or hygiene. Main intervention targets are sensory perception and processing, emotion and behavioral regulation, and development of autonomy. Parents are taught how to motivate their child to engage in social interaction and play without directly prompting or discouraging specific behaviors, and how to interpret their child’s emotional expressions in order to facilitate reciprocal interactions. Therapists also teach the parents how to transfer these skills to their home situation. Instruction of parents also includes psychoeducation, training of behavioral observation focusing on social signals, video analysis of play interactions, and hands-on coaching regarding play and everyday activities.

The initial intervention period is followed by 2 years of follow-up care by a FIAS therapist (8 h per month on average), during which the therapist provides regular coaching, analyzes the parents’ play interventions at home, and fosters exchange with other institutions involved, i.e. kindergarten, in order to follow the child’s development. In contrast with other early intervention programs that usually last from 1 to 2 years, FIAS focuses on a very high-intensity treatment period of about 100 h during the first 18 days, followed by less intensive follow-up care. During the follow-up period, the parents are encouraged to provide their child with play sessions of 1 to 2 h of per day in order to consolidate and amplify the gains made during the initial period [for details, see Herbrecht et al. (13)].

FIAS demands high motivation and commitment from parents during both the initial 18-day period at the treatment center and the continuing regular play sessions at home. In this respect, families participating in the FIAS treatment program represent a “positive selection”; not all mothers and fathers of children with ASD are able and/or willing to stay away from home and to suspend their professional and other customary activities for 18 days and nights in order to spend up to 6 h per day, including weekends, in the confines of the FIAS center. The particular conditions of FIAS also mean that it is not possible to include matched control groups receiving some form of sham treatment. Consequently, properly controlled clinical trials of FIAS treatment are not feasible.

### Participants

Between January 2011 and November 2018, 62 young children with autism according to DSM-IV/DSM-5 (14, 15) criteria were treated at the FIAS intervention center in Muttenz, Switzerland. By the end of June 2019, 32 of these children had full, 2-year follow-up data and these were the data used in the current analysis. The 32 children represent a sub-sample of the 40 patients whose 18-day outcomes have been described in a previous paper (Herbrecht et al., 2019). Three children having been treated at the FIAS center in 2011 had only 1 year of follow-up care, but still got their 2-year assessment. Diagnosis of ASD at baseline was based on direct observation and assessment by expert clinicians not involved in the subsequent interventions, using the Autism Diagnostic Observation Schedule (ADOS/ADOS-2 Module 1) and the Autism Diagnostic Interview-Revised ((ADI-R) (16); German version (17). None of the children had additional relevant neurological or somatic problems. All the families were currently living in Switzerland but were of various nationalities. All parents spoke either German or English. Parents of all children included in the study gave their written informed consent to the anonymized use of their data. During the 2-year follow-up period, according to their age, all children were integrated in regular or special education settings and followed additional occupational or speech therapy sessions.

### Measures

#### Autism Symptoms

The ADOS-2 is a semi-structured, standardized interaction and observation tool that assesses social communication and interaction as well as restricted and repetitive behaviors during child–adult interaction (11, 12). Different modules are administered depending on expressive verbal language ability and age. ADOS Module 1 for pre-verbal- to single-word-using children was used to assess all children at baseline. For the 1-year assessments, Module 1 was used in most cases; however, for one child Module 2, which requires simple phrase speech, was more appropriate. At the 2-year assessments, four children switched to ADOS Module 2, while one child (the one assessed using ADOS Module 2 at the 1-year assessment) switched to Module 3 which requires fluent speech.

The ADOS-2 provides a total score resulting from coding behaviors during the ADOS assessment according to a strict algorithm. For most assessments, the ADOS-2 version was used. If the prior ADOS-version had been used (i.e. baseline assessments before 2013), we calculated the updated ADOS-2 algorithms for comparability of all ADOS results. The ADOS-2 severity score is intended to allow comparisons across different ages, language abilities, and modules. In this study, both the ADOS total and severity scores are reported. In addition, assessments of autism spectrum-related symptoms provided a further categorization in terms of symptom levels: “high,” “moderate,” “low,” or “minimal-to-no-evidence.”

#### Level of Functioning

As in our previous studies (10, 18), we used an ASD-adapted version of the Children’s Global Assessment Scale, the DD-C-GAS (Developmental Disorders–Child–Global Assessment Scale) (19); German translation (20), as the reference measure of children’s level of functioning in daily life. The DD-C-GAS comprises 10 levels of functioning, using a 0 to 100 scale in four domains: everyday functioning, intellectual performance, communication, and social behavior. Scores below 70 indicate that a child has important special needs in that particular domain. DD-C-GAS assessments were made by clinicians, based on parents’ observations. In the current study, we used the DD-C-GAS as an independent instrument to assess changes in the four domains after the FIAS intervention. We also explored whether changes on this instrument corresponded to changes in ADOS scores. The DD-C-GAS ratings were made at baseline, directly after the 18-day intensive intervention period, at 1-year follow-up, and at 2-year follow-up. As this instrument was not used for all children (see below), the total number of DD-C-GAS data is smaller than the one of the ADOS.

#### Treatment Adherence

Adherence to treatment during follow-up intervention is crucial when non-clinicians are mediators of the intervention (3). During the 2 years of FIAS follow-up care, parents were regularly encouraged to provide play sessions of at least 1 to 2 h duration per day. To date, few studies have examined therapists’ persistence in training parents or other intervention mediators, or parents’ fidelity to the intervention. As follow-up care in the FIAS approach is notably long compared with the shorter, initial high-intensity period, we considered treatment fidelity during the 2 years of follow-up to be a relevant factor when interpreting the follow-up results. As part of FIAS procedures, the follow-up therapists allocated to each family were requested to provide an annual global judgment of parents’ treatment adherence (using the four categories of minimal, low, sufficient, and excellent), taking into consideration the parents’ observed and reported adherence to the therapists’ inputs and their active co-operation in implementing the learned strategies during follow-up.

### Procedures

Assessments were made at baseline and at 1 and 2 years after the initial intensive period (hereafter referred to as baseline, 1-year, and 2-year follow-up assessments), the 2-year time point coinciding with the end of the FIAS follow-up care. Autism symptom levels and follow-up levels of functioning were assessed by clinical psychologists at the special Autism Unit of the Child and Adolescent Psychiatry University Department in Basel. None of the evaluators was involved in the FIAS intervention. Baseline functioning levels were assessed by the lead FIAS therapist working with each child. Treatment adherence was evaluated by the follow-up reference therapist assigned to each family (see **Table 1** for an overview of the evaluation procedure).

**Table 1:** Evaluation procedures.

Assessment domain and instruments		Time point			Evaluator
		Base-line	1-yearfollow-up	2-yearfollow-up	
**Autism symptoms**	ADOS TS, SS	X	X	X	FIAS independent clinical psychologists
**Level of** **functioning**	DD-C-GAS	X			FIAS reference therapist
**Level of** **functioning**	DD-C-GAS		X	X	FIAS independent clinical psychologists
**Treatment adherence**			X	X	FIAS reference follow-up therapist

ADOS TS, ADOS Total Score; ADOS SS, ADOS Severity Score.

### Statistical Analyses

Data analysis was performed using non-parametric methods. Behavioral changes assessed by the ADOS and DD-C-GAS were evaluated using Friedman’s rank sum test for repeated measurements and Wilcoxon signed rank tests for comparisons between two time points. If the Friedman test indicated significance, Wilcoxon signed rank tests with adjustment for multiple testing by Bonferroni–Holm were performed between each pair of two time points. The outcome measures were changes in ADOS total and severity scores, the DD-C-GAS subdomain scores, autism symptom level, and treatment adherence scores. In order to evaluate the impact of either age or autism symptom severity at baseline on outcome parameters, analyses of change were performed by age category (<=43 months; >43 months) and baseline ADOS total score category (</=20; >20) respectively. The extent of relationships between the DD-C-GAS findings and the ADOS scores at the three time points, as well as between changes from baseline to 1- and 2-year follow-ups for both instruments and treatment adherence scores were assessed by Spearman’s rank correlation coefficients. The analysis was based on values as observed, except for one missing Year 1 ADOS observation which was replaced by the average of baseline and Year 2 values. The sensitivity analysis with imputed missing DD-C-GAS observations showed no relevant differences.

## Results

### Background and Demographic Data

We analyzed the data of 32 children (28 males, 4 females) who had been treated at the FIAS center between January 2011 and July 2017 and who had completed their 2-year follow-up assessment. The children’s mean age when the FIAS intervention started was 44.0 ± 8.3 months.

The mean ADOS total score at baseline was 20.0 ± 3.46, while the mean severity score was 7.0 ± 1.43 indicating moderate to high average levels of autism-related symptoms. Mean DD-C-GAS subdomain scores at baseline were similar for self-care (30.6 ± 14.21) and communication (31.0 ± 12.05), slightly lower for social behavior (27.0 ± 10.44), and higher for intellectual skills (42.1 ± 16.17). Scores at baseline indicated significant special needs in all four domains, especially in the areas of social behavior and communication.

After splitting the group into two according to median age (43 months), no relevant age-related differences were observed for any of the ADOS or DD-C-GAS variables at baseline (Mann-Whitney-U-test, data not shown), although ADOS severity scores were slightly higher in the older age group than in the younger group (7.4 ± 1.35 *vs.* 6.7 ± 1.45 respectively). We observed no differences in baseline scores between males and females. Because of the small number of females, we did not retain the sex category for analyzing changes in outcome parameters (see **Table 2**).

**Table 2:** Summary statistics of background and demographic data for all participants and by subgroups.

Parameter		Age		Sex		Baseline ADOS Total Score	
	All N = 32	≤43 mo. N = 17	>43 mo. N = 15	Male N = 28	Female N = 4	≤20 N = 17	>20 N = 15
**Age**							
mean (std)	44.0 (8.3)	37.9 (4.98)	50.9 (5.31)	44.1 (8.88)	43.5 (1.29)	42.5 (8.84)	45.8 (7.55)
med	43.0	39.0	48.0	42.5	43.5	42	44
min–max	25–60	25 –43	44–60	25–60	42–45	25–59	32–60
**Age category n (%)**							
≤43 mo.	17 (53.1)	–	–	15 (53.6)	2 (50.0)	10 (58.8)	7 (46.7)
>43 mo.	15 (46.9)	–	–	13 (46.4)	2 (50.0)	7 (41.2)	8 (53.3)
**ADOS Total score**							
mean (std)	20 (3.46)	19.4 (3.61)	20.7 (3.26)	20 (3.66)	19.8 (1.89)	17.1 (2.31)	22.9 (1.36)
med	20.5	20.0	22.0	20.5	20.5	17.0	23.0
min–max	11–26	11–24	16–26	11–26	17–21	11–20	21–26
**ADOS Severity score**							
mean (std)	7 (1.43)	6.7 (1.45)	7.4 (1.35)	7.1 (1.5)	6.5 (0.58)	5.8 (0.54)	8.3 (0.86)
med	6.5	6.0	8.0	6.5	6.5	6.0	8.0
min–max	4–10	4–9	6–10	4–10	6–7	4–6	7–10
		**Age**		**Sex**		**Baseline ADOS Total Score**	
	**All** **N = 31**	**≤43 mo**. **N = 17**	**>43 mo**. **N = 14**	**Male** **N = 27**	**Female** **N = 4**	**≤20** **N = 17**	**>20** **N = 14**
**DD-C-GAS Self-care**							
mean (std)	30.6 (14.21)	30.6 (15.08)	30.6 (13.65)	29.7 (11.34)	36.3 (29.26)	30.9 (12.55)	30.1 (16.49)
med	25.0	25.0	25.5	25.0	35.0	31.0	23.0
min–max	5–70	5–70	17–55	17–55	5–70	5–55	17–70
**DD-C-GAS Communication**							
mean (std)	31.0 (12.05)	32.5 (13.89)	29.1 (9.53)	31.3 (10.88)	29.3 (20.55)	32.1 (13.15)	29.7 (10.92)
med	29.0	32.0	28.5	29.0	31.0	30.0	25.0
min–max	5–55	5–55	17–51	17–55	5–50	5–51	17–55
**DD-C-GAS Social behavior**							
mean (std)	27.0 (10.44)	27.8 (11.74)	26.1 (8.96)	27.0 (9.43)	27.0 (17.87)	27.2 (12.28)	26.8 (8.11)
med	25.0	31.0	25.0	25.0	30.0	23.0	25.0
min–max	5–50	5–50	13–43	13–50	5–43	5–50	13–40
**DD-C-GAS Intellectual skills**							
mean (std)	42.1 (16.17)	39.1 (17.91)	45.7 (13.52)	42.2 (12.69)	41.8 (34.9)	44.7 (18.73)	38.9 (12.33)
med	41.0	35.0	43.0	41.0	40.5	50.0	38.5
min–max	5–81	5–65	21–81	19–65	5–81	5–81	21–60

No relevant group differences were observed for any of the ADOS or DD-C-GAS variables (Mann-Whitney-U test), except for the ADOS scores, as expected.

### Core Autism Symptom Outcomes

#### ADOS Findings by Time Point and Changes From Baseline

Mean ADOS total scores decreased from 20.0 ± 3.46 at baseline to 18.5 ± 5.91 at 1-year follow-up and statistically significantly to 17.1 ± 6.16 at 2-year follow-up (p = 0.031). Mean severity scores decreased numerically from 7.0 ± 1.43 at baseline to 6.8 ± 2.01 at 1-year follow-up and to 6.4 ± 1.83 at 2-year follow-up (see **Table 3**). The possible impact of age, total ADOS scores, and ADOS severity scores at baseline were estimated by comparing subgroups above and below the respective median values: no statistically significant or relevant subgroup differences were noted for any of these baseline variables (data not shown).

**Table 3:** Summary statistics for ADOS total and severity score by time point and change from baseline, and results of Friedman test and pairwise comparisons (N = 32).

				Friedman			Pairwise comparisons°	
	Baseline	Year 1	Year 2	Test	Bsl-Year 1	Bsl-Year 2	Bsl-Year 1	Bsl-Year 2
				p-value			p-value	p-value
ADOS Total score								
mean (std)	20 (3.46)	18.5 (5.91)	17.1 (6.16)	0.031*	1.5 (4.89)	2.9 (4.92)	0.171	0.006**
min–max	11–26	6–26	6–26		−6–13	−8–16		
ADOS Severity score								
mean (std)	7 (1.43)	6.8 (2.01)	6.4 (1.83)	0.236	0.2 (1.95)	0.6 (1.93)	n.a.	n.a.
min–max	4–10	3–10	2–10		−3–3	−5–5		

*p-value < 0.05; **p-value < 0.01. The significance level = 0.05.; °p-value from pairwise comparisons following significant Friedman test (Wilcoxon signed rank test adjusted by the Bonferroni–Holm correction for multiple testing. Bsl, baseline; n.a., not applicable.

#### Change in Autism Symptom Level

In addition to ADOS total and severity scores, level of autism spectrum-related symptoms could be further categorized (Lord et al., 2012): “high” encompasses severity scores from 8 to 10; “moderate,” scores from 5 to 7; “low,” scores from 3 to 4; and “minimal-to-no-evidence,” scores of less than 3. According to this categorization, in the present study 13 children had “high,” 18 had “moderate” and 1 had “low” levels of autism symptoms at baseline. In the older age group the percentage was 53 and 47% for high and moderate autism symptom levels respectively, while in the younger group the respective figures were 30 and 65%. From baseline to 2-year follow-up, all but two children showed no change or a decrease in these levels of autism symptoms: one of these children changed from low to high levels and the other from moderate to high levels of autism symptoms at the 2-year follow-up; 11 children showed a decrease in these levels at the 2-year follow-up: 5 changed from high to moderate, 1 from high to low, 5 changed from moderate to low. We did not observe any other relevant differences by age or ADOS total score category at baseline (see **Table 4**).

**Table 4:** Changes of ADOS symptom levels from baseline to Year 2.

ADOS symptom level autistic spectrum at baseline		ADOS symptom level autistic spectrum at Year 2		
		High	Moderate	Low
**High**	13	0	7	6
**Moderate**	18	5	6	7
**Low**	1	0	1	0
**Total**	32	5	14	13

### Developmental Functioning Outcomes

#### DD-C-GAS Findings by Time Point and Changes From Baseline

**Figure 1:**
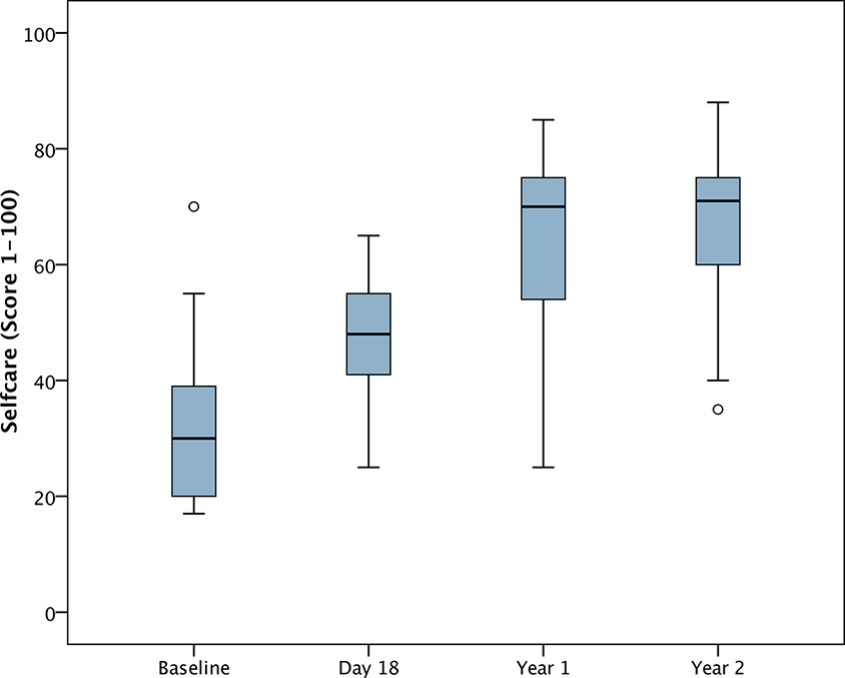
Box plot: DD-C-GAS Self-care (N = 25). Non-parametric repeated measures ANOVA (Friedman test): p = 0.000***. Pairwise comparisons between time points (Wilcoxon signed rank test): p-values adjusted by Bonferroni-Holm were 0.000***, except for comparison Year 1 to Year 2 (p = 0.060).

**Figure 2:**
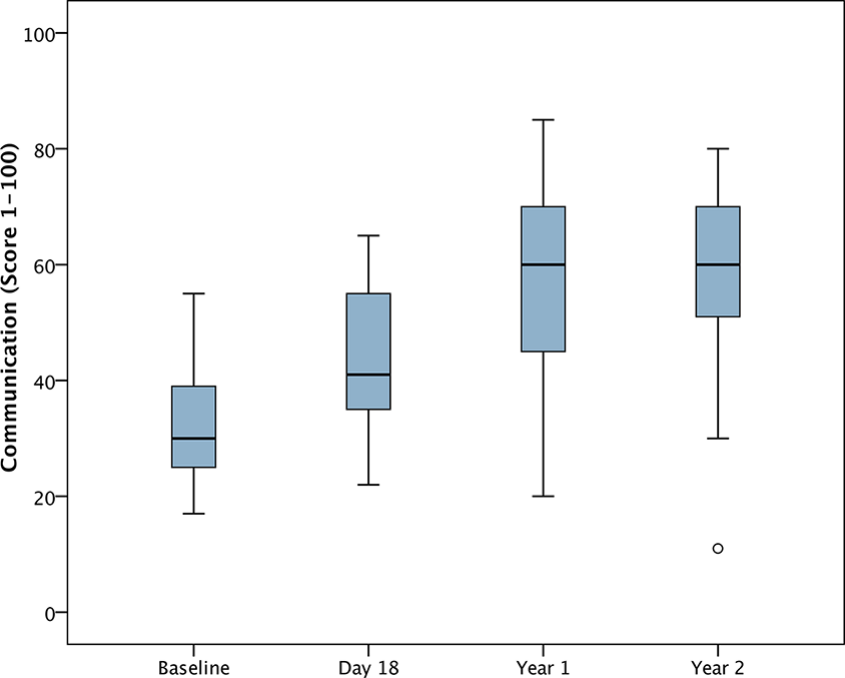
Box plot: DD-C-GAS Communication (N = 25). Non-parametric repeated measures ANOVA (Friedman test): p = 0.000***. Pairwise comparisons between time points (Wilcoxon signed rank test): p-values adjusted by Bonferroni-Holm were 0.000***, except for comparisons Day 18 to Year 1 (p = 0.002**), and Year 1 to Year 2 (p = 0.460).

**Figure 3:**
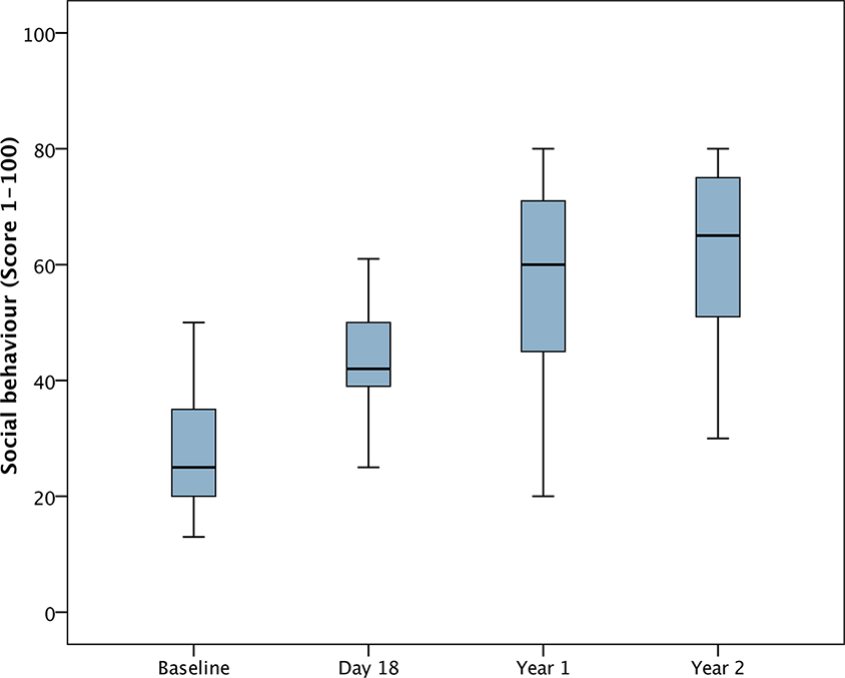
Box plot: DD-C-GAS Social behavior (N = 25). Non-parametric repeated measures ANOVA (Friedman test): p = 0.000***. Pairwise comparisons between time points (Wilcoxon signed rank test): p-values adjusted by Bonferroni–Holm were 0.000***, except for comparison Day 18 to Year 1 (p = 0.002**), Year 1 to Year 2 (p = 0.145).

**Figure 4:**
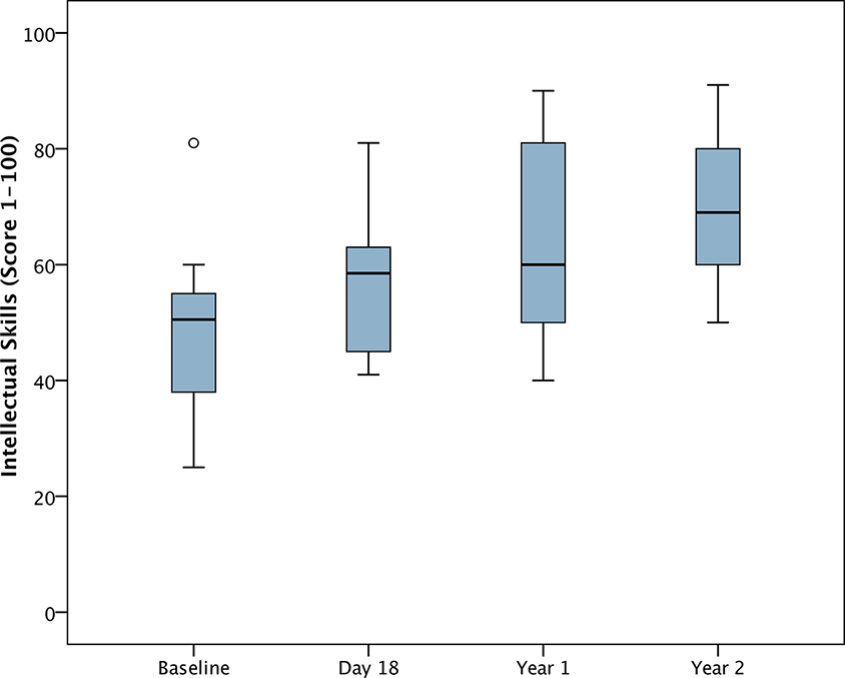
Box plot: DD-C-GAS Intellectual skills—Subjects with all time points (N = 18). Non-parametric repeated measures ANOVA (Friedman test): p = 0.000***. Pairwise comparisons between time points (Wilcoxon signed rank test): p-values adjusted by Bonferroni–Holm were 0.000***for comparison Baseline (Bsl) to Year 2, <0.01** for comparisons Bsl to Day 18 and Year 1, and Day 18 to Year 2, <0.05* for Day 18 to Year 1 and Year 1 to Year 2.

For the analysis of changes from baseline, DD-C-GAS data from 25 children were available for all time points. Seven parents were unable to evaluate the subdomain intellectual level for their child. There were highly statistically significant changes (i.e. improvements) in all four DD-C-GAS domains from baseline to 1-year follow-up, as well as from baseline to 2-year-follow-up (see **Figures 1****–****4**). DD-C-GAS mean scores increased by an average of about 30 points, with relatively small changes between the 1- and 2-year follow-ups.

At baseline we observed slightly higher median scores in the younger age group compared with the older group for the domains of communication and social behavior. Changes over time were also somewhat greater in the younger age group, except for the domain of communication where changes were greater in the older group. There was a trend towards greater improvement on the DD-C-GAS intellectual skills domain in the lower ADOS total score category at baseline.

**Figure 5:**
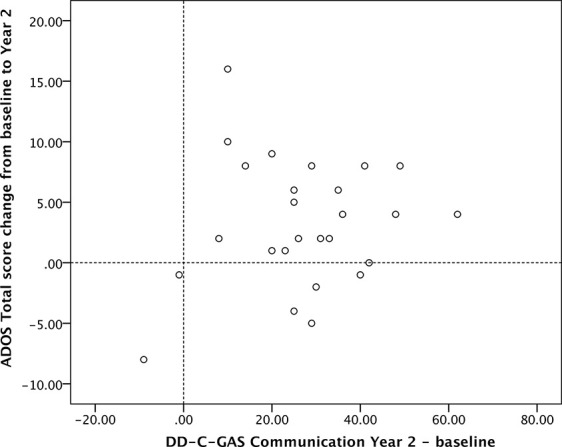
Scatterplot: ADOS Total score change versus DD-C-GAS Communication change (N = 26). Changes for ADOS scores were calculated as differences baseline – Year 2 to present improvements as positive values.

**Figure 6:**
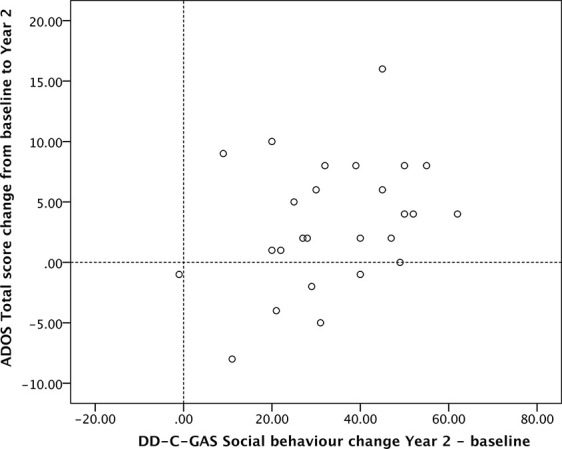
Scatterplot ADOS Total score change versus DD-C-GAS Social behavior change (N = 26). Changes for ADOS scores were calculated as differences baseline – Year 2 to present improvements as positive values.

DD-C-GAS subdomain scores were highly intercorrelated. We observed moderately strong negative correlations between ADOS total scores and DD-C-GAS subdomain scores at baseline. At 1-year follow-up, there were highly significant negative correlations for all subdomains except self-care. At 2-year follow-up, negative correlations were moderately significant for ADOS total score and all DD-C-GAS subdomain scores (data not shown). In contrast, there were no relevant correlations between the two instruments in terms of change from baseline. Scatterplots of changes in ADOS total as well as ADOS severity scores *versus* DD-C-GAS subdomains illustrate the heterogeneity of the individual results (see **Figures 5** and **6**). Nevertheless, the vast majority of data points were consistently found in the upper right quadrant of all the scatterplots, indicating positive changes of varying magnitude in the behavioral and symptomatic areas investigated (DD-C-GAS and ADOS).

### Treatment Adherence

Treatment adherence was rated as excellent in 18 of the 32 families at 1-year follow-up and in 14 of 29 families at 2-year follow-up (ratings were not done for three families treated in 2011 whose follow-up care lasted one year only). Treatment adherence was rated as being minimal in three families at one-year and in four families at 2-year follow-up. There was a trend towards lower treatment adherence in the older age group compared with the younger age group, and overall, treatment adherence decreased slightly over time in both age groups. When analyzed by ADOS total scores at baseline, treatment adherence was significantly higher in the lower-score group at 1-year follow-up and still so, albeit less prominently, at 2-year follow-up (**Table 5**).

**Table 5:** Summary statistics of treatment adherence findings, by baseline ADOS total score category.

Baseline ADOS Total score category	≤20 N = 16					>20 N = 16				
Treatment adherence	Minimal	Low	Sufficient	Excellent	Total	Minimal	Low	Sufficient	Excellent	Total
Year 1 - n (%)	0 (0.0)	2 (12.5)	2 (12.5)	12 (75.0)	16	3 (18.8)	1 (6.3)	6 (37.5)	6 (37.5)	16
Year 2 - n (%)	1 (7.1)	2 (14.3)	2 (14.3)	9 (64.3)	14	3 (20.0)	4 (26.7)	3 (20.0)	5 (33.3)	15

Correlations between treatment adherence and ADOS total scores were statistically significant (p < 0.01) at both 1-year and 2-year follow-up (r = −0.45, r = −0.60). Correlations between treatment adherence and ADOS severity scores were statistically significant (p < 0.01) at 2-year follow-up (r = −0.50). Significant positive correlations were found between treatment adherence and DD-C-GAS subdomain scores at 1-year follow-up (between 0.41 for intellectual skills, p < 0.05; and 0.50 for selfcare, p < 0.001). At 2-year follow-up, correlations were mostly low and not significant (see **Table 6**).

**Table 6:** Correlations between treatment adherence, ADOS scores, and DD-C-GAS findings at both Year 1 and Year 2.

Spearman-rho			ADOSTotal score	ADOSSeverity score	DD-C-GASSelf-care	DD-C-GAS Communication	DD-C-GASSocial behavior	DD-C-GAS Intellectual skills
**Year 1**	**Treatment adherence**	**Corr. Coeff**.	−0.452^**^	−0.338	0.501**	0.483**	0.489**	0.411*
		**N**	32	32	28	28	28	24
**Year 2**	**Treatment adherence**	**Corr. Coeff**.	−0.595^**^	−0.504^**^	0.370	0.129	0.245	0.167
		**N**	29	29	26	26	26	20

*Correlation significant at level 0.05 (two-sided); **Correlation significant at level 0.01 (two-sided).

## Discussion

The primary objective of the current study was to examine whether symptomatic and behavioral changes in young children with ASD were sustained 1 and 2 years after an initial 18 days of intensive FIAS intervention. The 2-year time point corresponded to the end of the FIAS follow-up care for 29 of the 32 children. In an earlier study (10) involving most of the current patient sample, we demonstrated that short-term changes during the initial intervention period could be sensitively captured by means of the Autism Behavior Coding System (ABCS), a video-based instrument for assessing core autism symptoms during therapist–child interactions. The current study focused on longer-term outcomes 1 and 2 years after the initial intervention period, as reflected in different levels of outcome and their potential correspondence.

Baseline ADOS total and severity scores indicated moderate to high autism symptom levels in 18 and 13 children, respectively, while DD-C-GAS scores were suggestive of major deficits in all behavioral subdomains, most clearly in social behavior, as assessed by parents. No relevant age- or sex-related differences were observed for any of the ADOS or DD-C-GAS variables at baseline.

We first analyzed changes over time in core autism symptoms as assessed by experienced ASD clinicians who had not been involved in the intervention. ADOS total and severity scores decreased from baseline to 1-year and 2-year follow-up, the change in ADOS total score from baseline to 2-year follow-up reaching statistical significance, indicating a continuous and relevant overall decrease in core autism symptoms. This finding is of importance, as the ADOS was not primarily designed to be sensitive to intervention-related changes in autism symptoms. For that reason, many other post-intensive intervention long-term follow-up studies did not use the ADOS and did not demonstrate a reduction in core autism symptoms; instead, many concentrated on outcome measures concerning IQ and adaptive behavior (4). Some of the studies that did use the ADOS as an outcome measure also showed symptom reduction following intensive intervention, as reflected in changes in ADOS severity scores (see **Table 7** for an overview).

**Table 7:** Early intervention studies reporting post-treatment changes in ADOS scores.

Study	Personimplementing	Duration (weeks)	Intervention hours/week	NIG/CGBaseline	NIG/CGFollow-up	MeanAge (years)	IGADOS-SSPre-/post-intervention	CGADOS-SSPre-/post intervention
Aldred et al., (21)	P	48	12	14/14	14/14	4.2	*16.1/11.8	*15.6/16.1
Dawson et al. (22)	C/P	96	30	24/24	24/21	4.3	7.2/6.5	6.9/7.3
Estes et al., (2)	C	96	20	24/24	21/18	6	*6.9/5.8	*7.5/7.3
Pickles et al., (23)	C/P	48	12	77/75	59/62	3.6	8/7.3	7.9/7.8
Rogers, et al. (24)	C/P	96	24	55/63	45/36	2	7.2/6.69	7.83/6.19
Kitzerow et al. (25)	C	48	2	20/20	20/18	5.5	7.3/5.95	6.5/6.1
Robain et al. (26)	C	52	1–12 (CG) 18–22 (IG)	22/38	22/38	3.0	8.14/6.73	7.13/7.18

C, Clinician; P, Parent; IG, Intervention Group; CG, Control group; SS, Severity Score; *, Total score.

The decrease in ADOS severity scores reported in previous studies varies from 0.2 (22) to 1.4 points (26). Most of the studies summarized in **Table 7** reported on ADOS severity scores, except for the study by Aldred, Green, and Adams (21) which showed a decrease for ADOS total scores. Interestingly, an evaluation of a 2-year intervention using the Early Start Denver Model (ESDM) revealed no relevant effect on core autism symptoms immediately after treatment (22) but positive findings at a follow-up assessment 2 years later (2). Unfortunately, statistical details on the reduction in autism symptoms are not available from this study. A recent publication by Rogers et al. (24) reported no relevant changes nor differences in autism symptom severity between an ESDM intervention and a community treatment group over a 2-year intervention period. Conversely, a study of a 12-month, pre-school, parent-mediated autism communication intervention (Preschool Autism Communication Treatment, PACT) found a significant decrease in autism symptoms (21). The first study to show longer-term autism symptom reduction was that by Pickles et al. (23), who re-evaluated patients from an initial PACT study nearly 6 years after the intervention. At this time point, children who had received PACT still maintained some improvement: their ADOS Severity Score values were down to 7.3 ± 2.0 from 8.0 ± 1.4 points at baseline, whereas those who had been on TAU (treatment as usual) had virtually the same ADOS Severity scores at baseline (7.9 ± 1.4) and at follow-up (7.8 ± 1.8). A very recent publication by Robain et al. (26) revealed positive changes in autism symptom severity as well as of cognitive abilities in an intensive intervention group (following the Early Start Denver Model approach) compared to a control group including different community treatment settings available in the greater Geneva area.

The results from the DD-C-GAS showed highly significant improvements on all subdomains, these changes being numerically more impressive than the results obtained with the ADOS. Overall, DD-C-GAS scores increased by approximately 30 points (within a total range of 100 points), whereas the mean decrease in the ADOS total score was about three points (within a range of 28 points) and around one point in the severity score (within a range of 10 points).

To interpret these findings, one first needs to consider that the four DD-C-GAS subdomains are not independent of one another but are highly intercorrelated. When considering the numerical disparity between the changes in the two scales one should also bear in mind two further aspects. First, the ADOS was administered under narrowly defined conditions, i.e. within approximately 45 min of assessment in a novel environment by a clinician who was initially unknown to the child and was not involved in the treatment. In this regard, the ADOS could be viewed as an objective assessment instrument administered under conditions that were unlikely to lead to an overestimation of the child’s capacities. In contrast, the DD-C-GAS ratings were based on information provided by the child’s parents, who live and interact daily with the child and who are most likely influenced by their hopes and expectations of positive intervention effects as well as by a generally positive attitude towards their own intervention. This phenomenon of performance bias is ubiquitous although, conversely, caregiver reports are considered to have superior external validity (27). Second, ADOS ratings represent a current, moment-based observation of autism symptoms, whereas the DD-G-GAS includes observations and reports from a longer period, converted into a clinician’s rating.

We also investigated whether and how the findings using the ADOS corresponded to the more global behavioral assessments based on the DD-C-GAS. There were mostly non-significant (and as expected, negative) correlations between the two instruments at baseline, yet highly significant negative correlations at 1- as well as 2-year follow-up, i.e. cross-sectionally at three separate time points. In contrast, there were no meaningful correlations between *score changes* observed over time, except for the changes between ADOS total score and the DD-C-GAS subdomain social behavior (p < 0.01) at 1-year follow-up. At 2-year follow-up, the changes between these two scores remained relatively high, but did not reach statistical significance. As pointed out before, the two instruments imply different metrics of assessment, different sources of information and assessment conditions, different observation periods and areas of observation, as well as a different potential bias in favor of the intervention. Given these differences, one might assume that the changes seen on the two scales are both clinically relevant but relatively independent of one another. Nevertheless, and importantly, most children’s combined outcome scores were located in the upper right quadrant of the scatterplots presented in **Figures 5** and **6**, indicating improvement in autism symptoms *and* level of functioning domains.

Changes on both scales were virtually independent of age and severity of autism symptoms at baseline, suggesting that older and more severely affected children may benefit to a similar extent as younger children do from a FIAS intervention. Although the small sample size in the current study does not permit any general conclusion with regard to the optimal age range for an intensive intervention such as FIAS, our observations are in line with the results of a recent meta-analysis that included 29 early intervention studies (3). These authors found the largest effect sizes for communication outcomes at the age of 3.8 years i.e. almost identical with the median value of the children in our sample (43 months). Estes et al. (2) found long-term effects 2 years after the end of an ESDM intervention which children of less than 30 months of age at baseline, suggesting the effectiveness of early intervention approaches in very young children.

As the parents were instructed by FIAS therapists to continue to provide regular play sessions under their supervision during the 2 years of follow-up, we considered treatment adherence to be of crucial interest in terms of intervention outcome. The findings of a recent meta-analysis (3) suggest that the benefit of early intervention is greatest when it is conducted by clinicians but may also be significant when implemented by parents. The authors observed that both the fidelity of clinicians in teaching parents and the fidelity of parents in ultimately providing the intervention in everyday life were of crucial importance to the overall outcome. In the current study, treatment adherence in the 2 years following the intensive 18-days of in-house FIAS treatment was evaluated by the reference therapist for each family. In support of the conclusions drawn by Fuller & Kaiser (3) we found a close and mostly statistically significant relationship between parents’ treatment adherence and the ADOS total and severity scores, indicating that the better parents’ treatment adherence was, the lower the children scored on the ADOS at 1- and 2-year follow-up. Interestingly, correlations became even higher at 2-year follow-up. Overall, treatment adherence was satisfactory in our sample even though a slight decrease over time was observed. Detailed evaluation of treatment adherence at the level of each family revealed a relevant factor of the decrease: as children grew older, the use of external support facilities, such as kindergarten or special education services as well as additional therapies, became more time-consuming. Thus, regular, daily play sessions between parents and their children in time may have become more difficult to accomplish. The relationship between treatment adherence and ADOS scores seems to be more meaningful than the one between treatment adherence and DD-C-GAS subdomain scores. This may be due to the fact that both, treatment adherence and the ADOS, are external measures assessed by independent clinicians, whereas the DD-C-GAS is based on the parents’ reports. As stated earlier, the special conditions of the FIAS approach do not allow a matched control group to be included in a study. FIAS demands high motivation and commitment from parents, as well as intensive, active involvement in the intervention both for the initial period of 18 days at the treatment center and for continuing regular play sessions at home. These factors cannot be emulated in a credible control group.

**Table 7** provides core findings of seven published controlled intensive intervention trials with young ASD children lasting between 48 and 96 weeks. In three of these studies no relevant positive changes in either ADOS Severity or Total Scores were observed in the control groups, and three studies reported slightly negative changes on TAU; the only paper reporting positive changes of ADOS Severity Scores on TAU was the one by Rogers et al. (24). However, this study differs from the others in at least two relevant respects: The children included were younger (average 2 years) than the ones in the other studies (average between 3.6 and 6 years), and the average improvement observed in the control group was numerically higher than in the group treated according to the Denver model. A very recent paper by Robain et al. (26) reported no positive changes in a TAU control group. This study is of particular interest for a number of reasons: (a) the patient sample is comparable to ours in terms of size and average age of the children in the control TAU group; (b) like ours, the study was performed in Switzerland, and it is reasonable to assume that the families involved in it lived under similar socio-economic circumstances like the ones participating in the FIAS study; (c) availability and use of additional treatments (behavioral, educational) is comparable in Swiss urban and suburban areas such as Geneva (26) and Basel (FIAS study). In summary, there is no evidence from controlled studies that TAU groups show positive changes on a comprehensive clinical scale such as the ADOS. Nevertheless, the changes we observed in favor of an intervention benefit cannot be specifically attributed to the FIAS treatment, as both maturation and influences such as family- and other support-related variables may have affected the children’s development. As Robain et al. (26) stated, children in either early intervention as well as in community treatment settings usually receive multiple interventions, yet specifically addressing social communication development may be crucial to improve core autism symptoms. This may explain the advantage of early intensive intervention approaches in improving core autism symptoms compared to nonspecific interventions reflected in control group settings as TAU.

In conclusion, the results of the current study suggest that the FIAS approach, which consists of an initial short and highly intensive 18-day intervention period followed by 2 years of parent-mediated follow-up care, had a positive impact on the core autism symptoms as well as the adaptive functioning of the children analyzed. Changes assessed with the more global clinical scale DD-C-GAS were numerically more impressive, but were also more likely to be biased by parents’ expectations, than were the results from the ADOS scale. The reduction in core autism symptoms and the higher level of functioning were found to be independent of age and autism severity at the start of the intervention.

## Data Availability Statement

The raw data supporting the conclusions of this article will be made available by the authors, without undue reservation.

## Ethics Statement

Written informed consent was obtained from the parents of all children included in the study. All procedures performed in studies involving human participants were in accordance with the ethical standards of the institutional and/or the National Research Committee, as well as with the 1964 Declaration of Helsinki and its later amendments or comparable ethical standards.

## Author Contributions

EH, MN, and RS designed the study. EH, OL, and EK acquired the data. MN, EH, OL, EK, KS, and RS analyzed and interpreted the data. EH, MN, and RS drafted the manuscript. All authors contributed to the article and approved the submitted version.

## Funding

OL was partially funded by the Universitäre Psychiatrische Kliniken (UPK) Basel Forschungsfonds, and by the Fondation Botnar.

## Conflict of Interest

RSis the President of the FIAS Foundation, a charitable, non-profit organization supporting the FIAS treatment center financially.

The remaining authors declare that the research was conducted in the absence of any commercial or financial relationships that could be construed as a potential conflict of interest.
